# Undifferentiated pleomorphic sarcoma in the anterior mediastinum: a case report and literature review

**DOI:** 10.3389/fonc.2024.1445149

**Published:** 2024-10-15

**Authors:** Luyao Wang, Anyu Xie, Luqin Ke, Pingfan Jia, Yuru Li, Xing Guo

**Affiliations:** Department of Radiology, Heping Hospital Affiliated to Changzhi Medical College, Changzhi, Shanxi, China

**Keywords:** undifferentiated sarcoma, anterior mediastinal tumor, computed tomography, magnetic resonance imaging, diagnosis

## Abstract

Primary undifferentiated high-grade pleomorphic sarcoma (UPS) in the mediastinum is exceptionally rare. This paper reports a unique case of anterior mediastinal UPS in an 84-year-old Asian male presenting with recurrent upper respiratory tract infections and upper abdominal discomfort. Imaging via CT and MRI suggested an invasive thymoma, but postoperative pathology confirmed UPS. Despite radical surgery, local recurrence occurred within three months, and palliative radiotherapy was ineffective. This case provides the first comprehensive imaging data of UPS in the anterior mediastinum, aiming to improve diagnostic accuracy for clinicians and radiologists by summarizing imaging features across various modalities.

## 1 Introduction

Undifferentiated pleomorphic sarcoma (UPS) is a diagnosis of exclusion that requires the elimination of various other tumor types prior to confirmation. Radiology plays a crucial role in initial diagnosis, long-term follow-up, and the evaluation of many treatment-related complications. Undifferentiated pleomorphic sarcoma (UPS) is a rare and aggressive mesenchymal malignancy without a clear cell of origin, previously known as malignant fibrous histiocytoma. It is a high-grade sarcoma without any specific differentiation, appearing at any age but most commonly between 50 and 70 years, with a slight male predominance, primarily originating from the soft tissue of the limbs (1). Histologically, it is characterized by pleomorphic and spindle-shaped tumor cells and a spiral growth pattern (2). However, it lacks clear diagnostic criteria and is easily confused with other mediastinal tumors. To date, imaging characteristics of UPS at different sites have been documented to enhance diagnostic efficiency, but less so for mediastinal cases; we report a case of anterior mediastinal UPS with complete imaging data.

## 2 Case presentation

**Figure 1:**
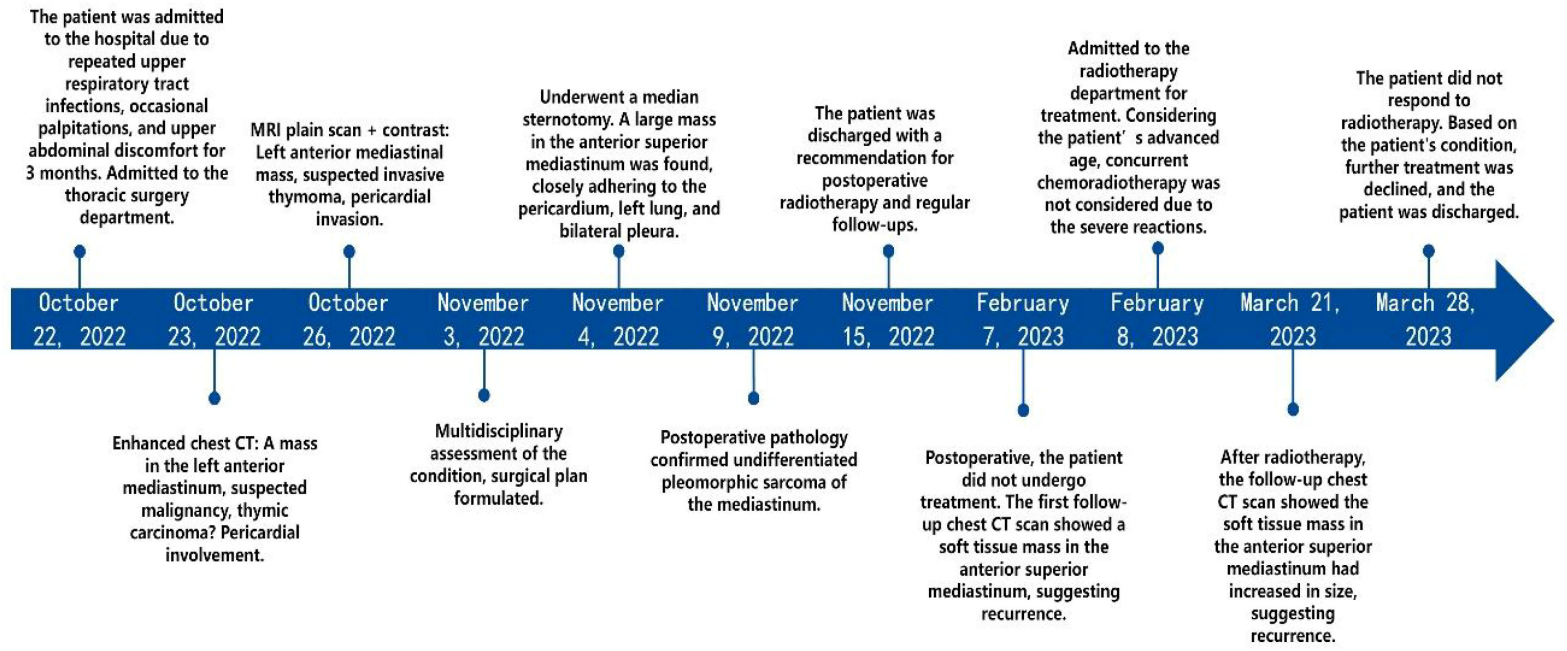
Timeline of the patient’s case.

Patient’s medical history: An 84-year-old Asian male presented with recurring upper respiratory infections, occasional palpitations, and upper abdominal discomfort over the last month (**Figure 1**). He has a 7-year history of hypertension, with blood pressure reaching as high as 180/100 mmHg. He had no known history of smoking, alcohol use, or recreational drug use. Over ten years ago, he underwent laparoscopic surgery for a renal cyst. Physical examination upon admission showed jugular vein distention and diminished breath sounds in the upper right lung. Laboratory tests revealed: CD3 + 42.24% (reference range 56%–86%), helper/inducer T-cells (CD3+CD4+) 23.08% (reference range 33%–58%), and NK cells (CD3-CD56+) 38.75% (reference range 5%–26%). Blood tests indicated hemoglobin at 70 g/L (normal range 120–160 g/L), indicating moderate anemia.

### 2.1 Auxiliary examinations

**Figure 2:**
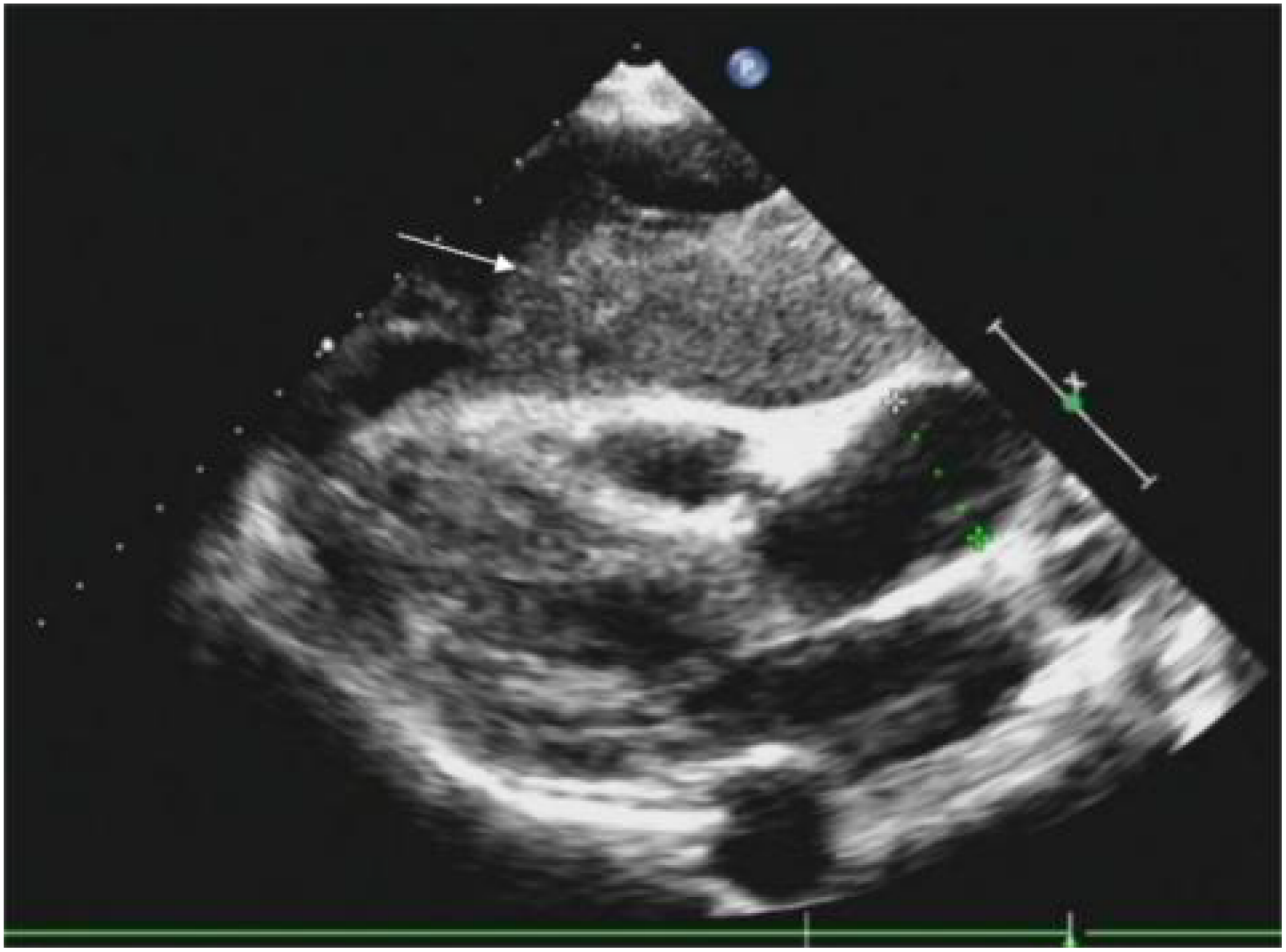
A hypoechoic mass is visible on the outer pericardium, with indistinct boundaries with the anterior wall of the right ventricle (indicated by a white arrow), causing posterior displacement of the heart.

**Figure 3:**
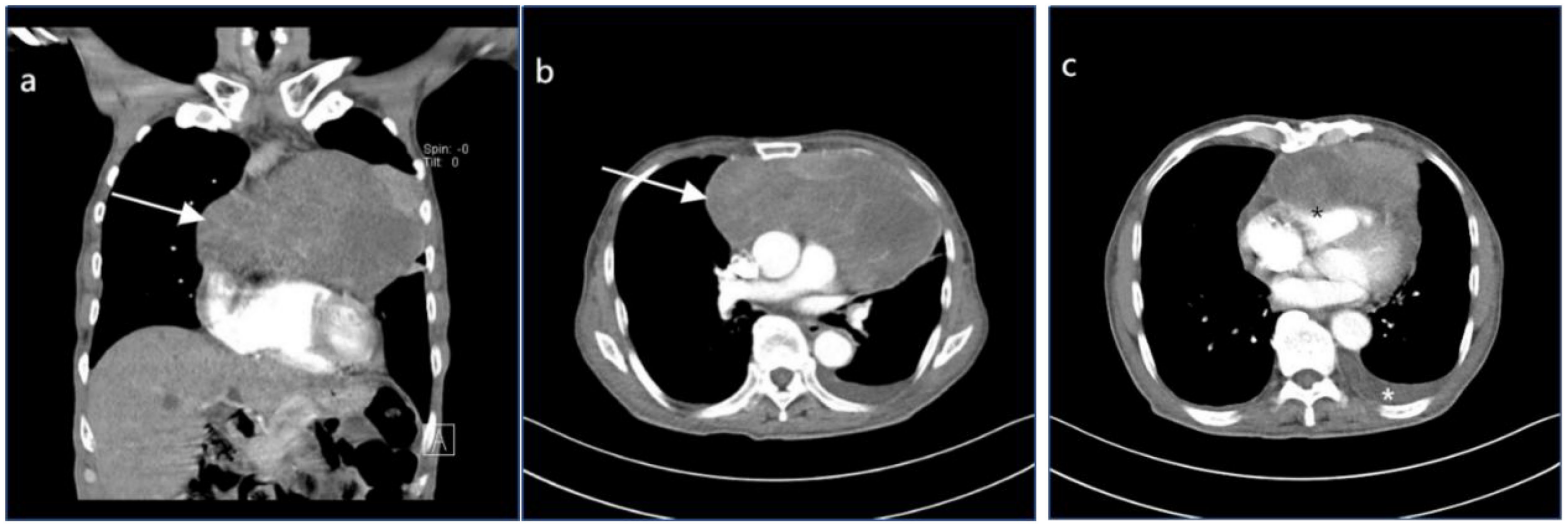
**(A, B)** demonstrate enhanced chest CT scans in the coronal and transverse planes, respectively. An irregular mass is noted in the left anterior mediastinum (highlighted by white arrows), with an approximate cross-sectional size of 165mm×96mm, showing heterogeneous enhancement with CT values ranging approximately from 20 to 61 Hounsfield units (HU). **(C)** illustrates the lesion invading the pericardium with indistinct margins (denoted by black asterisks), accompanied by posterior displacement of the cardiac great vessels and presence of left pleural effusion (denoted by white asterisks).

**Figure 4:**
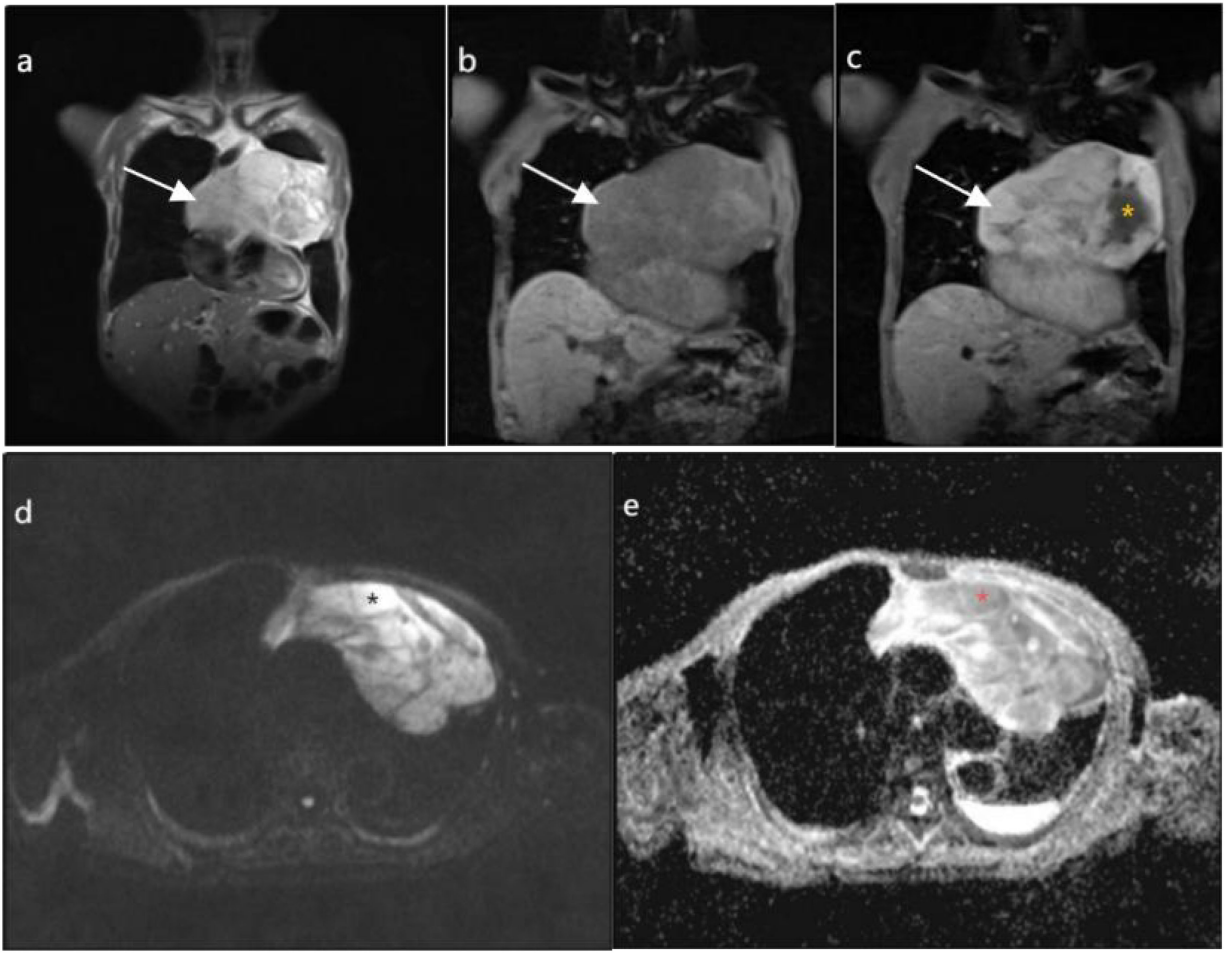
**(A, B)** depict coronal T2-weighted imaging (T2WI) and T1-weighted imaging (T1WI), respectively, demonstrating an irregular mass in the anterior mediastinum (highlighted by white arrows), displaying mixed T2 and T1 signal intensities with internal low signal septations on T2WI, measuring approximately 167mm×97mm×106mm with well-defined margins. **(C)**, on coronal T1WI post-contrast imaging, reveals heterogeneous enhancement with irregular cystic necrotic areas within (denoted by yellow asterisks). **(D)** shows diffusion-weighted imaging (DWI) with a b value of 800, demonstrating diffusely high signal intensity in the solid component (denoted by black asterisks). **(E)** displays an apparent diffusion coefficient (ADC) value of 1.430×10-3mm2/s (denoted by red asterisks).

Routine ultrasound identified a hypoechoic mass located near the pericardium, displaying indistinct boundaries with the anterior wall of the right ventricle and resulting in the posterior displacement of the heart (**Figure 2**). CT scan revealed an irregular mass in the left anterior mediastinum measuring approximately 16.5 cm × 9.6 cm, with heterogeneous enhancement and a CT value range of approximately 20 to 61 HU. The mass exhibited involvement of the pericardium with uncertain demarcations, leading to the displacement of major cardiac vessels and the presence of left pleural effusion (**Figure 3**). MRI imaging depicted an irregular mass in the anterior mediastinum with mixed T2 and T1 signals, measuring around 16.7 cm × 9.7 cm × 10.6 cm, and displaying well-defined margins. DWI demonstrated high signal diffusion, with an ADC value of 1.430 × 10-3 mm²/s, indicating areas of irregular liquefactive necrosis within the mass. DWI showed high signal diffusion, with an ADC value of 1.430 × 10-3 mm²/s, and irregular liquefied necrotic areas were seen. The boundary of the lesion with the pericardium was indistinct, and there was posterior displacement of the major cardiac vessels (**Figure 4**).

### 2.2 Surgical procedure

Via a median sternotomy, a large mass was observed in the upper anterior mediastinum, predominantly on the left side, measuring approximately 20 cm × 15 cm × 5.0 cm. The mass was firm in texture, lobulated, and partially tense, adhering closely to the pericardium, the upper lobe of the left lung, and bilateral mediastinal pleura. It was carefully dissected and completely excised along with portions of the mediastinal pleura and pericardium.

### 2.3 Postoperative pathology findings

**Figure 5:**
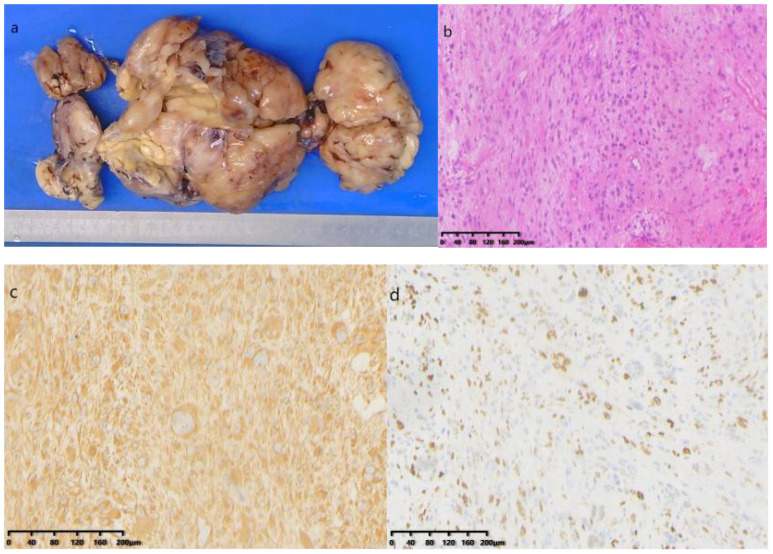
**(A)** Gross specimen: A collection of gray-white, gray-yellow, and gray-brown tissue; **(B)** Hematoxylin and Eosin staining microscopic examination: The tumor consists of morphologically diverse and large atypical cells, with large, deeply stained nuclei, prominent nucleoli, and occasional pathological mitotic figures and tumor giant cells; **(C)** Immunohistochemical staining shows positive for vimentin; **(D)** Ki-67 positive in 50% of cells (HE×200).

Postoperative Pathology Findings: Gross examination (**Figure 5A**): A mass of gray-white, gray-yellow, and gray-brown tissues, total volume 25 cm × 16 cm × 6.5 cm, with areas of gray-white translucency and others of gray-yellow and gray-brown, solid and of medium consistency. Pathological results suggested a soft tissue tumor, pending further immunohistochemical diagnosis. Immunohistochemistry (**Figures 5B, C**): Immunostaining showed positive results for vimentin Ki-67 (50%) and negative results for SMA, Des, MyoD1, Myogenin, S-100, MDM2, CKpan, CD34, SOX-10, HMB-45, EMA. CD163, Thus, the condition was diagnosed as undifferentiated pleomorphic sarcoma.

### 2.4 Postoperative follow-up

**Figure 6:**
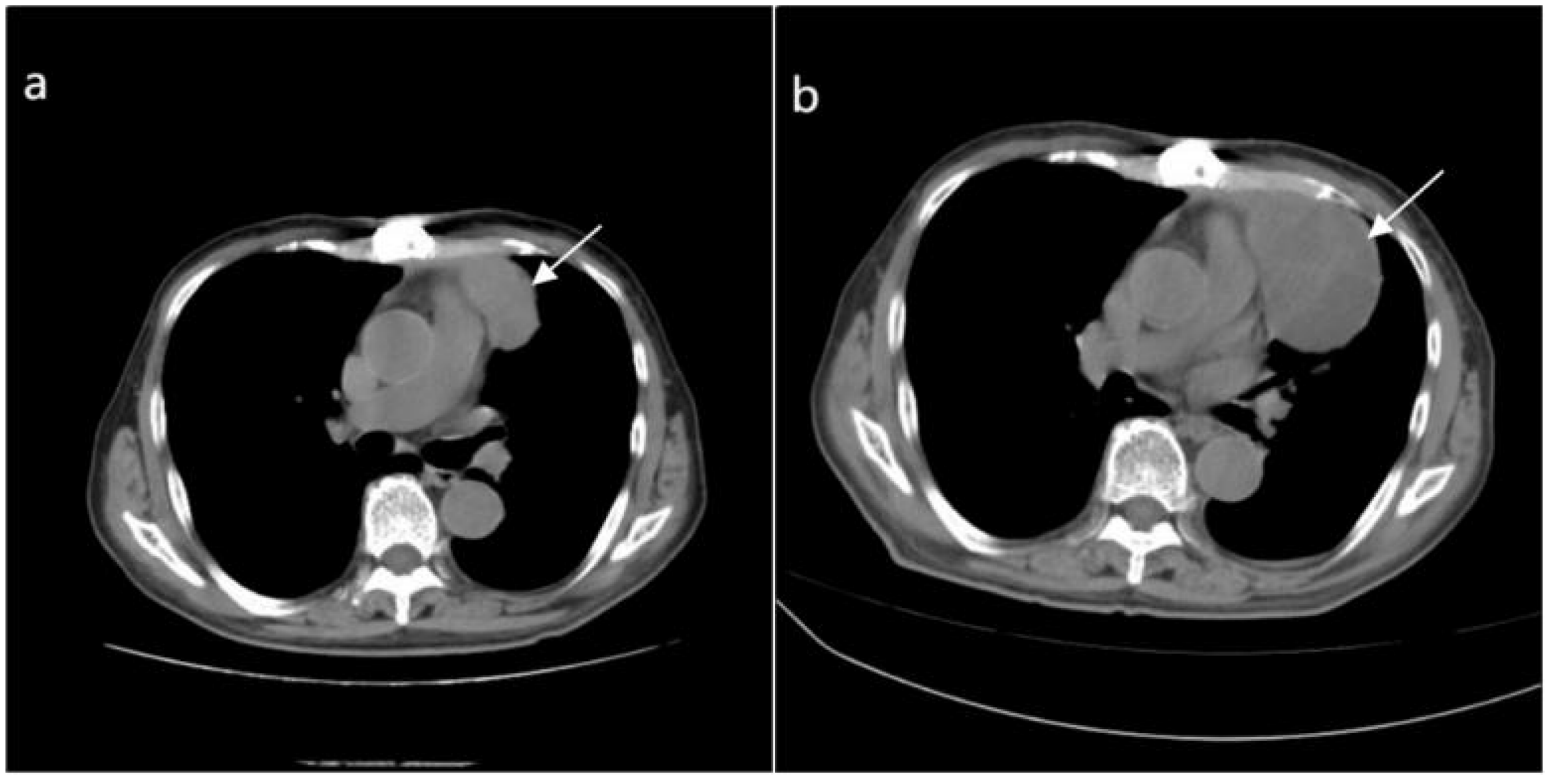
**(A)** Depicts an irregular soft tissue density shadow in the upper anterior mediastinum (highlighted by white arrows), approximately 32mmX51mmx80mm in size, observed 3 months after surgery for the anterior mediastinal mass. **(B)** Taken 2 months after radiotherapy, a larger irregular soft tissue density shadow is visible in the upper anterior mediastinum (highlighted by white arrows), measuring approximately 56mmx78mmx92mm, with uneven density and showing enlargement compared to the previous image.

The patient recovered well post-surgery, with stable condition, and there was no particular discomfort. Postoperative radiotherapy and regular check-ups were recommended. Three months later, a follow-up CT scan of the chest revealed an irregular soft tissue density in the upper anterior mediastinum, measuring about 3.2 cm × 5.1 cm × 8.0 cm, suggesting a recurrence (**Figure 6A**). The patient was readmitted to our radiotherapy department. Chemotherapy was not considered for the time being due to the patient’s advanced age and the obvious side effects of receiving radiotherapy. A chest CT scan two months following radiation therapy revealed that the tumor had gotten bigger (**Figure 6B**). Given the tumor’s insensitivity to radiotherapy and poor therapeutic effects, the patient and family were informed, and he requested discharge after the completion of radiotherapy, refusing further treatment.

## 3 Discussion

Undifferentiated sarcoma, previously known as malignant fibrous histiocytoma (MFH), is a rare mesenchymal-origin soft tissue malignancy clinically. The concept of undifferentiated sarcoma was introduced in the 2013 WHO classification of soft tissue tumors, distinguishing it into four subtypes under microscopy: pleomorphic, spindle cell, round cell, and epithelioid (3). The WHO classification was updated in 2020, categorizing it into three subtypes: undifferentiated pleomorphic sarcoma, undifferentiated spindle cell sarcoma, and undifferentiated round cell sarcoma (4). Weiss et al (5). analyzed 200 cases of MFH, reporting it most commonly occurs in males and individuals over 40, with an incidence rate of one per 100,000. Due to the small number of published case reports, diagnosing UPS remains challenging.

To further explore UPS, we searched the literature in Embase, Cochrane, and PubMed, with a date range of 1 January 2013 to 21 February 2024 considered for the studies. The search criteria were as follows: undifferentiated pleomorphic sarcoma [Title/Abstract]. Notably, the World Health Organization has chosen to replace this term with a neutral and more accurate label (i.e. UPS) (3). Studies were included based on the following criteria: (1) primary UPS, histologically confirmed; (2) detailed imaging data; (3) case reports. Detailed imaging data were defined as textual information given according to at least 2 of the following criteria: tumor size, borders, aggressiveness, hemorrhage, necrosis, calcification, contrast enhancement (CT or MRI), MR signal behavior (T1W or T2W), and ultrasound performance.

**Figure 7:**
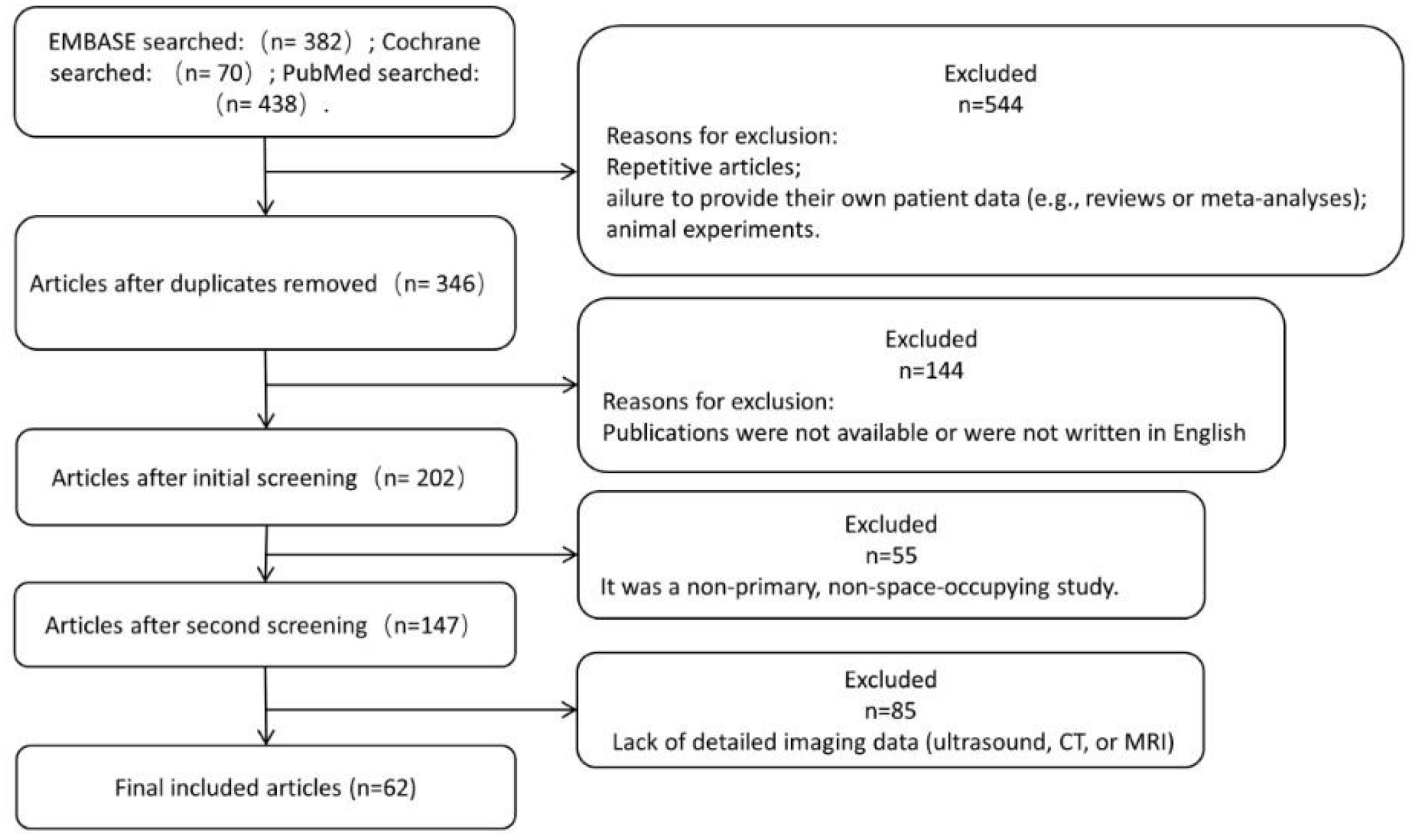
Flow chart.

A total of 382 EMBASE, 70 Cochrane and 438 PubMed studies published between 1 January 2013 and 21 February 2024 were identified. Duplicate articles, failure to provide own patient data (e.g. reviews or meta-analyses), animal studies were excluded; publications that were not accessible, or not written in English were excluded from our analyses; non-primary, non-occupying UPS was not considered for further analysis; the lack of detailed imaging data (CT or MRI) in publications led to exclusion, and ultimately 62 publications were eligible for further analysis (**Figure 7**).

**Figure 8:**
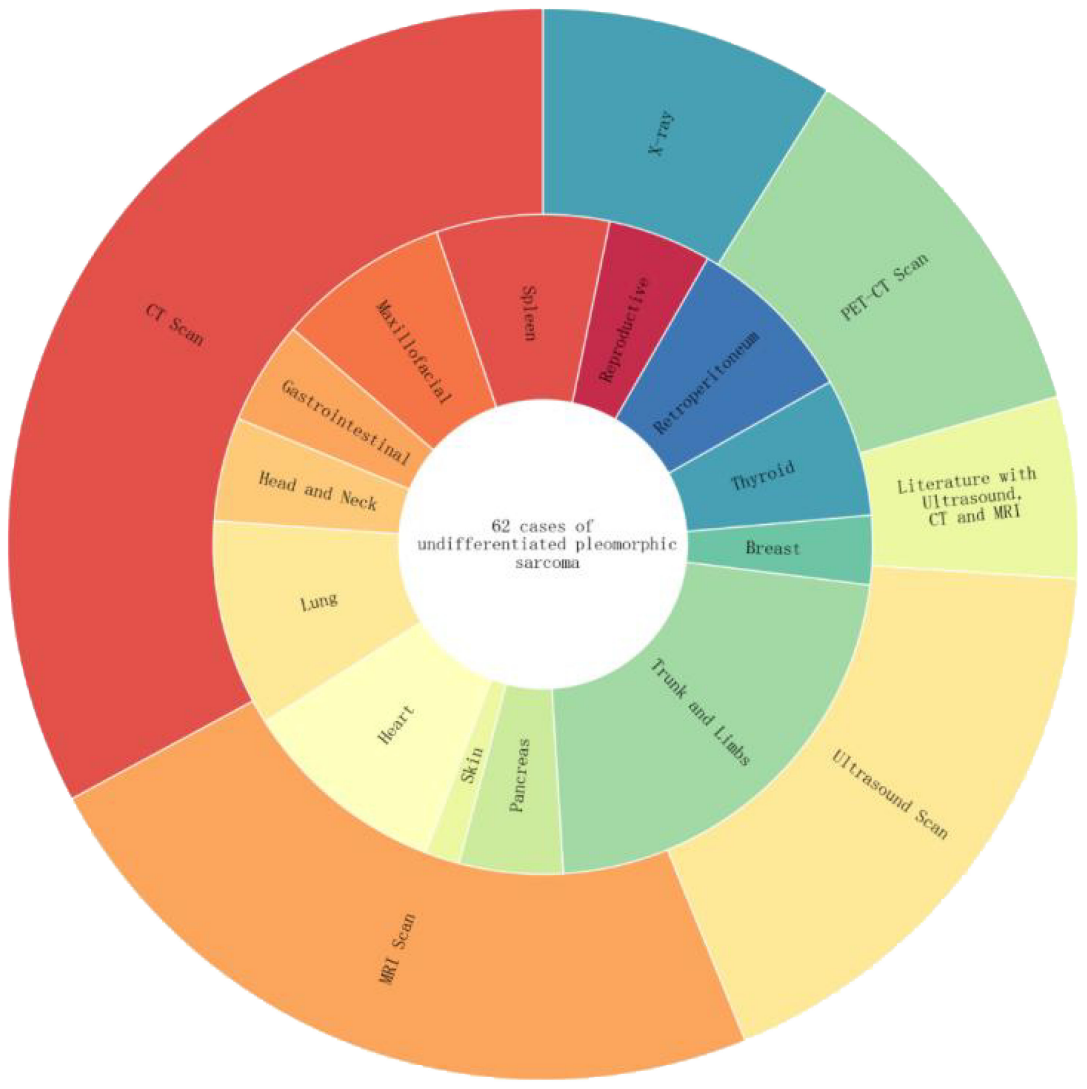
The inner circle represents the percentage distribution of UPS cases in different body regions. For example, the trunk and limbs have the highest proportion at 13/62. The outer circle represents the distribution of imaging examination techniques among the 62 UPS cases, including CT, MRI, ultrasound, PET-CT, and cases utilizing a combination of ultrasound, CT, and MRI imaging techniques.

After searching and screening, UPS was distributed in different parts of the body, with a total of 62 articles (**Figure 8**): soft tissues of the limbs and trunk (13 cases), thyroid (4 cases), gastrointestinal tract (5 cases), heart (6 cases), pancreas (3 cases), spleen (3 cases), breast (2 cases), maxillofacial area (5 cases), retroperitoneum (5 cases), lungs (3 cases), head and neck (3 cases), reproductive organs (3 cases), skin (1 case), and mediastinum (6 cases). Diagnostic examinations included X-rays (13 cases); CT scans (48 cases); MRI scans (34 cases); ultrasound examinations (26 cases); PET-CT scans (17 cases).

From initial diagnosis, staging, prognosis and stratification, to identifying locally invasive disease and distant metastases, radiological investigations are crucial for patients with sarcomas. X-rays are usually used for initial evaluation (especially in the extremities, chest) and can show calcification, translucency (corresponding to fat) and associated bone changes. Ultrasound is the preferred initial method for superficial lesions due to its accessibility and excellent spatial and contrast resolution, allowing differentiation between parenchymal and cystic lesions and revealing internal morphology and vascularity. CT is commonly used to assess lesions in the head and neck, mediastinum and retroperitoneal regions, and contrast-enhanced scans providing better delineation of tumor vasculature and malignant potential. Any lesion that is indeterminate or suspicious by ultrasound or CT, deep tumor, superficial tumor invading the fascia, incompletely visible tumor by ultrasound, tumor >5cm should be further investigated using magnetic resonance imaging (MRI) (6).

Contrast-enhanced MRI is considered the optimal modality for characterizing soft tissue tumors (STTs). MRI can delineate tumor contents (or matrix), tumor margins, and surrounding tissues, especially for heterogeneous tumors, necrotic content, and hemorrhagic components. Following successful surgical resection with clear margins, patients should undergo continuous monitoring by CT or MRI for recurrence or late metastasis (7). Although PET imaging is not routinely used in the initial staging of pleomorphic sarcomas (8), PET/CT, as a metabolic imaging modality, can identify necrotic areas of high-grade sarcomas because of their increased FDG uptake can determine the systemic impact of UPS. It can also assess the systemic impact of undifferentiated pleomorphic sarcoma (UPS), which may be difficult to detect with conventional CT and MRI, thereby predicting overall survival and disease-free survival in patients (9, 10).

**Figure 9:**
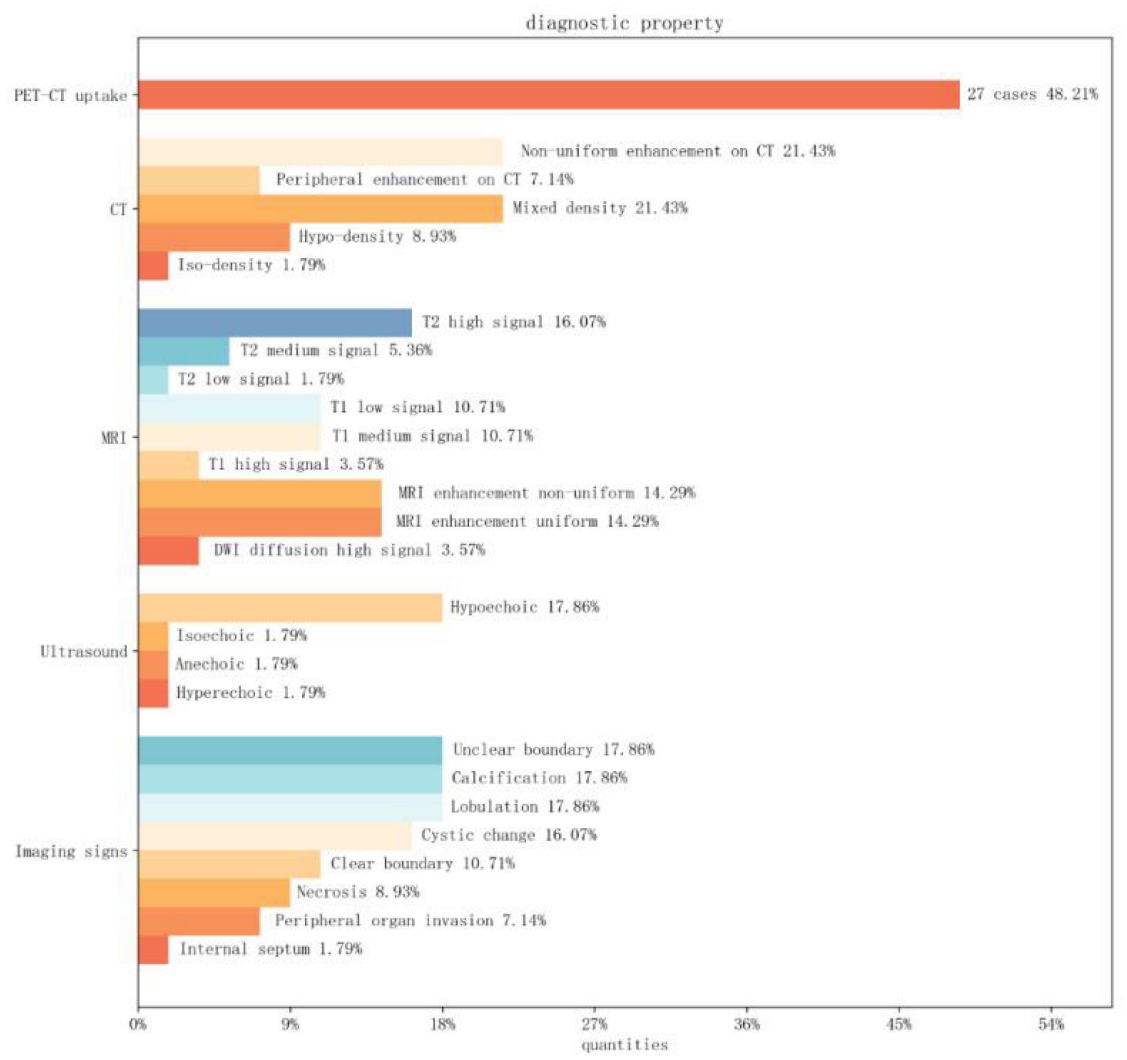
Image Feature Bar Chart: The vertical axis represents PET-CT, CT, MRI, ultrasound findings, and special imaging characteristics. The horizontal axis represents the percentage distribution among cases of non-mediastinal UPS.

Non-mediastinal UPS tumors (56 cases) median diameter (max-min) 6.4 cm (1.8-2.4 cm).16% (9/56) tumors were hypoechoic ultrasonographically. On CT they were usually heterogeneous in appearance.17% (10/56) tumors were lobulated and 26% (15/56) The tumor contained areas of necrosis or cystic degeneration and hemorrhage within the tumor, with calcification in a few areas, most commonly in the thyroid gland.17% (10/56) of the tumors had unclear borders, and tumor enhancement on enhancement was heterogeneous or peripherally enhanced.3% (10/56) of the tumors had a heterogeneous appearance on CT. On MRI, the signal intensity varied according to the internal components of the tumour.10% (6/56) showed predominantly low or intermediate signal on T1WI, with a few high signals, and 16% (9/56) showed predominantly high or mixed signals on T2WI, with intratumorally cystic necrotic areas of varying sizes. The one case of a lesion in the pancreas demonstrated low-signal segregation of T2 signals, and most of them showed inhomogeneous enhancement on enhancement. The one case of UPS in the soft tissue of the tibia showed enhancement tailing forward along the fascia. Only 3% (2/56) of the literature mentioned restricted diffusion-weighted imaging (DWI), with one instance providing an apparent diffusion coefficient (ADC) value of 1.210 × 10^−3 mm^2/s (11). PET-CT exhibited high uptake in 48% (27/56) of cases, with specific numerical values mentioned in 5 instances (**Figure 9**).

Reports on all cases in the mediastinum (12–17) indicate that UPS patients ranged in age from 50-84 years, with an average age of 68.1 years, and a male to female ratio of 4:3 (**Table 1**). Five cases were located in the anterior mediastinum, including one concurrent with thymoma and another in the middle mediastinum. The average tumor diameter was 12.5 cm. CT scans showed soft tissue density in six cases, irregular morphology in five cases, uneven enhancement in two cases, and invasion of peripheral tissues in four cases. Five tumors caused pleural and pericardial effusions. This case is the only preoperative MRI examination in the mediastinal cases, showing mixed T2 and T1 signals, with internal low signal separations on T2WI, high signal on DWI, and an ADC value of 1.430×10-3 mm2/s, consistent with the MRI characteristics of UPS in the pancreas, retroperitoneum, and stomach (9, 18, 19).

**Table 1:** Clinical and imaging data of seven patients with pleomorphic undifferentiated sarcoma in the mediastinal site.

	First author/s, years	Sex/age, years	Tumor site	Tumor size, (cm)	Immunohistochemistr	Imaging Features		Pericardium/pleural effusion	Therapy	Clinical outcome	(Refs.)
1	Sato et al.,2020	F/77	Anterior Mediastinum	NA	Vim (+)		Soft tissue density; No Obvious enhancement observed	Bilateral pleuraleffusion	Radical operation was performed for recurrence insitu after radical operation	Died of respiratory failure in 33 days	(11)
2	Xiaodan Chen et al., 2023	F/67	Anterior Mediastinum and Thymus	3.8	Vim (+), Ki-67 (50%+), BcL-2 (+), P16(+)		Soft tissue density; Clear boundary; Uneven progressive enhancement	NA	Thoracoscopic enlarged thymectomy + thoracoscopic partial pericardiectomy	Recurrence or metastasis was not reported at 6-month follow-up	(12)
3	Okuda et al., 2015	M/56	Anterior Mediastinum	13	Vim (+), Ki-67 (54.7% +), WT-1 (+), NSE (+)		Irregular shape; Soft tissue density; Invasion of left lung, pericardium and lung hilum	NA	Tumor resection + left lung resection; pericardium partial resection + nerve and lymph node resection	Recurrence occurred within 1 month after the operation; recurrent hemorrhage occurred within 3 months; and death occurred within 4 months	(13)
4	Nakayama et al.,2019	F/82	Middle Mediastinum	NA	NA		Irregular shape; Soft tissue density	NA	Palliative Treatment	Died of right heart failure within 1 month	(14)
5	Matsumoto et al.,2021	M/50	Anterior Mediastinum	16	Almost all of the tumor markers were negative		Irregular shape; Pericardium invasion	Pericardial effusion and pleural effusion	NA	Died a few days later	(15)
6	Hu et al.,2023	M/61	Anterior Mediastinum	10	CKpan, CAM5.2, EMA, Vimentin, Ki67 (+)		Irregular shape; Soft tissue density; The anterior chest wall, pericardium, and liver capsule were invaded	right pleural effusion	Epirubicin 130 mg combined with tislelizumab 200 mg Q3W was given for 6 cycles	Died 8.5 months later	(16)
7	Present study	M/84	Anterior Mediastinum	20	Vimentin(+), Ki-67(50%+)		Irregular shape; Soft tissue density; The boundary is not clear; Non-uniform enhancement; Pericardium invasion	left pleural effusion	Radical surgery and radiotherapy were performed	Died 3.5 months later	

NA, Not Applicable.

Tumors in the mediastinum requiring differential diagnosis include thymic epithelial tumors, germ cell tumors, lymphomas, and neuroendocrine carcinomas (20). Thymic epithelial tumors, most commonly thymomas, appear on CT as well-demarcated round or oval soft tissue masses within the thymus region. The tumors typically present with soft tissue attenuation and mild to moderate contrast enhancement, CT shows lobulated or irregular contours or heterogeneous enhancement suggestive of infiltrating thymoma or thymic carcinoma (21). Occasionally, focal areas of low attenuation are found within the tumor reflecting hemorrhage, necrosis or cyst formation, and linear or circumferential calcifications are occasionally seen in pericyclic thymomas and invasive thymomas (22). On MRI, thymoma shows moderate signal intensity slightly higher than muscle on T1-weighted images and high signal intensity on T2-weighted images (23). Additionally, the absence of a strong capsule on MRI strongly suggests tumor invasion of surrounding tissues. Jeong et al. (24) reported that major vessel infiltration is an important characteristic of thymic tumors as it is only seen in thymic carcinoma. In this study case, the smooth contour of the anterior mediastinal irregular mass, the presence of septations, and the absence of vascular infiltration serve as differential points from thymoma/carcinoma, although both exhibit malignant signs similar to those of surrounding tissue structures, making differentiation challenging.

Lymphoma, a malignancy originating in the lymph nodes or lymphatic tissue, typically presents on CT as a large, irregular-shaped or conglomerate nodular soft tissue mass in the anterior mediastinum with unclear margins and uniform density. Mediastinal lymph node involvement is common, and approximately half of the cases have pleural and/or pericardial effusions (25). After enhancement, the mass often exhibits moderate, uniform enhancement and commonly envelops surrounding blood vessels without invading the vascular structures, presenting a “vascular encasement sign,” a characteristic imaging feature useful for differential diagnosis. Before treatment, mediastinal lymphomas typically do not show calcifications, an important consideration in differential diagnosis.

Germ cell tumors are composed of three types: teratomas, seminomas, and non-seminomatous malignant germ cell tumors. Over 80% of germ cell tumors are benign, with the majority being mature teratomas (26). Seminomas, the most common malignant subtype, display well-defined, lobulated or multinodular masses with relatively uniform signal intensity on MR images. Cystic changes, necrosis, and hemorrhage are visible but usually localized, with thin septations within the mass. On contrast-enhanced MRI, seminomas typically show uniform enhancement of the mass. Mediastinal teratomas vary in appearance on CT and MRI based on their contents. On CT, teratomas most commonly appear as well-demarcated unilocular or multilocular cystic lesions containing fluid, soft tissue, and fat attenuation. Various forms of calcification might also be present, occasionally including teeth or bone. However, the capsules of teratomas are characteristically thickened, whereas the capsules of other mediastinal cystic lesions are usually thin (27).

Thymic neuroendocrine tumors are commonly found in the anterior superior mediastinum and often present on CT as large soft tissue masses with cystic changes and necrosis, the extent of which is typically proportional to the tumor diameter. The radiological features on CT and MRI do not significantly differ from those of thymomas (28). Shimamoto et al. (29) reviewed the CT and MRI findings of 11 cases of thymic carcinoids (3 typical and 8 atypical carcinoids), reporting that most lesions had irregular margins and lacked a peripheral capsule. The masses were heterogeneous with necrotic or cystic changes and hemorrhage, and septa within the mass were rare.

The primary differential diagnoses for UPS are the multiple subtypes of STS. The most common differential diagnoses considered for UPS are Lipoma, Liposarcoma, Angiosarcoma, Angioma, Leiomyosarcoma, Osteosarcoma, and Dermatofibrosarcoma Protuberans. Tumor metastases from other sites are also considered (8).

The cornerstone for the management of UPS occurring in the trunk, head, neck, and limbs is complete and utter surgical resection with adequate free margins. This can be successfully accomplished via wide local resection of a free margin of 2 cm. Radiotherapy implemented after surgical excision is applied either when the free margins are distanced <1 cm from the lesion, the lesion invaded the bony structures, or when there are significant vascular or nerve entities involved (30). Furthermore, chemotherapy can also be considered for advanced UPS which has an advanced stage that hinders surgical resection or spread out in the body.

The gold standard for establishing a final diagnosis relies on competent histopathological analysis. Upon examination of the UPS specimens under the light microscope, it portrays vivid cellular atypia, prominent mitotic structures, and pleomorphic cellular components. The malignant lesion of UPS could also demonstrate a sheet-like, storiform, or fascicular tissue organization inside a fibrous-based stroma (31).

Nevertheless, the ultimate diagnosis is defined via thorough immunohistochemical analysis (32). Some of the IHC markers used to differentiate UPS from other STS include CD68, CD30, S100, SMA, Kinases, Desmin, Vimentin, Ki-67 proliferation index, and p53 (33, 34).

In recent years, genomic approaches have significantly advanced the identification of predictive biomarkers in soft tissue sarcomas (STS), improving differential diagnosis, prognosis assessment, and targeted therapy. Next-generation sequencing (NGS), as a pivotal tool, enables comprehensive tumor genomic profiling, providing the foundation for personalized treatment strategies. For instance, NGS can detect critical mutations and gene fusions that are essential for the treatment of various STS subtypes. Gounder et al (32). found through targeted gene sequencing that up to 31.7% of patients harbor actionable genetic alterations, which are of significant value in diagnosis and treatment decision-making. Simon et al (35). further analyzed the genomic characteristics of undifferentiated pleomorphic sarcoma (UPS) using NGS, identifying tumor mutational burden (TMB), microsatellite instability (MSI), and DNA damage repair defects as biomarkers that could predict responses to immunotherapy or targeted therapy. Moreover, NGS can identify high-risk genetic variants associated with cancer susceptibility, aiding in early screening and monitoring. Vanni et al (36). emphasized that combining genomics with pharmacology can assist in identifying predictive biomarkers, particularly for differential diagnosis, prognosis assessment, and targeted therapy in UPS. RNA sequencing analysis revealed that matrix metalloproteinase 13 (MMP13) and WNT7B genes are upregulated in UPS, which are linked to enhanced cell migration and tumor aggressiveness. Additionally, the immune system plays a crucial role in UPS chemosensitivity, suggesting potential directions for future immunotherapy. While it remains challenging to implement NGS for every sarcoma patient, the application of NGS in genetically complex sarcomas with limited therapeutic options offers the potential for precision medicine in patient subgroups, especially through emerging therapies such as immune checkpoint inhibitors.

This study had some limitations. First, undifferentiated pleomorphic sarcoma (UPS) in the anterior mediastinum is an extremely rare malignancy with a low incidence rate and typically lacks characteristic clinical presentations. Second, definitive diagnosis still relies on histopathology and immunohistochemistry for an exclusionary comprehensive diagnosis. Third, although genomic approaches, such as next-generation sequencing (NGS), have shown promise in identifying potential biomarkers and molecular targets for treatment, the rarity of UPS limits the availability of large genomic datasets specific to this malignancy. As a result, comprehensive genomic profiling and the identification of specific actionable mutations or pathways remain challenging. This limitation hinders the development of targeted therapies, and personalized treatment strategies for UPS based on genomic data are still in their early stages.

Regular surveillance for affected patients should be conducted thoroughly to ensure that there aren’t any relapses, recurrences, or metastases. With regards to clinical examination, it must be carried out at three-to-six-month intervals for the first 2 years postoperatively and then performed at an annual rate (37).

## 4 Conclusion

Primary undifferentiated high-grade pleomorphic sarcoma (UPS) in the anterior mediastinum is extremely rare, and imaging and pathological diagnosis are crucial. Although genomic approaches show potential, personalized treatment for UPS is still in its early stages, and future research and data collection will drive therapeutic advancements.

## 5 Patient perspective

The patient and their family, after careful consideration and on the advice of the doctors, jointly decided to proceed with radiation therapy. Due to the patient’s advanced age and the progression of the disease, they opted to forgo chemotherapy. The family expressed that their hope was to ensure the patient’s comfort and dignity in their final days, providing the best possible care and support. aggressive nature.

## Data Availability

The original contributions presented in the study are included in the article/supplementary material. Further inquiries can be directed to the corresponding author.
